# Crystal structure of human lysosomal acid lipase and its implications in cholesteryl ester storage disease[S]

**DOI:** 10.1194/jlr.RA120000748

**Published:** 2020-06-01

**Authors:** Francis Rajamohan, Allan R. Reyes, Meihua Tu, Nicole L. Nedoma, Lise R. Hoth, Adam G. Schwaid, Ravi G. Kurumbail, Jessica Ward, Seungil Han

**Affiliations:** * Pfizer Worldwide Research, Groton, CT 06340; † Pfizer Worldwide Research, Cambridge, MA 02139

**Keywords:** lysosomal acid lipase deficiency, Wolman’s disease, lipid metabolism, protein aggregation, molecular dynamic simulations

## Abstract

Lysosomal acid lipase (LAL) is a serine hydrolase that hydrolyzes cholesteryl ester (CE) and TGs delivered to the lysosomes into free cholesterol and fatty acids. LAL deficiency due to mutations in the LAL gene (*LIPA*) results in accumulation of TGs and cholesterol esters in various tissues of the body leading to pathological conditions such as Wolman’s disease and CE storage disease (CESD). Here, we present the first crystal structure of recombinant human LAL (HLAL) to 2.6 Å resolution in its closed form. The crystal structure was enabled by mutating three of the six potential glycosylation sites. The overall structure of HLAL closely resembles that of the evolutionarily related human gastric lipase (HGL). It consists of a core domain belonging to the classical α/β hydrolase-fold family with a classical catalytic triad (Ser-153, His-353, Asp-324), an oxyanion hole, and a “cap” domain, which regulates substrate entry to the catalytic site. Most significant structural differences between HLAL and HGL exist at the lid region. Deletion of the short helix, ^238^NLCFLLC^244^, at the lid region implied a possible role in regulating the highly hydrophobic substrate binding site from self-oligomerization during interfacial activation. We also performed molecular dynamic simulations of dog gastric lipase (lid-open form) and HLAL to gain insights and speculated a possible role of the human mutant, H274Y, leading to CESD.

Lysosomal acid lipase (LAL) is a serine hydrolase enzyme located in the lysosome, where it breaks down cholesteryl esters (CEs) and TGs into free cholesterol and fatty acids (1–3). LAL belongs to a gene family of mammalian acid lipases that includes gastric and lingual lipases (4). The enzyme is synthesized by almost all cells except erythrocytes. Defective LAL activity/diminished acid lipase have been associated with two autosomal recessive traits in humans: Wolman’s disease (WD) and CE storage disease (CESD) (5, 6). However, the clinical and biochemical phenotypes manifested by WD and CESD are significantly different. WD is an early onset disease that primarily affects infants and results in demise in the first three to twelve months of life (7). It is associated with severe liver complications, malabsorption, and growth failure (7). In contrast, CESD is a late onset form of the disease that occurs in both children and adults with residual (1% to ∼5%) LAL activity (8–10). The progressive accumulation of lysosomal CE and TG in CESD patients leads to elevated levels of serum transaminase and LDL cholesterol, and ultimately to liver failure, hyperlipidemia, and accelerated atherosclerotic diseases (8, 11, 12). The molecular mechanisms of these LAL loss-of-function mutants leading to WD and CESD have yet to be explored. Current treatment options for CESD phenotypes are limited to diets that exclude cholesterol- and TG-rich food, cholesterol-lowering drugs such as statins, and ultimately liver transplantation. Recombinant LAL replacement therapy has been shown to be effective in animal models and human clinical trials. Recently, Kanuma (sebelipase alfa) (Alexion Pharmaceuticals, Cheshire, CT) has obtained marketing authorization for the treatment of CESD in Europe and the United States with recombinant human LAL (HLAL) (13–15).

The gene (*LIPA*) that encodes HLAL was mapped on chromosome 10q23.2-q23.3, and it consists of 10 exons spread over ∼36 kb DNA (16). It shares 58% and 57% amino acid identity with human gastric lipase (HGL) and rat lingual lipase (RLL), respectively (4). HLAL cDNA encodes a 378 amino acid mature protein following the cleavage of 21 amino acids signaling peptide, with an expected molecular mass of 43 kDa. It contains six predicted glycosylation sites, three of which (Asn-15, Asn-80, and Asn-252; mature HLAL numbering) are highly conserved among members of the lipase gene family (17). Previous studies have suggested that the signal peptide sequence and cotranslational/posttranslational glycosylation modifications are important for LAL expression, secretion, and trafficking (17, 18). A cluster of highly conserved Cys residues, Cys-227, Cys-236, and Cys-244 (mature HLAL numbering), is present in all three enzymes (HLAL, HGL, and RLL). Human LAL contains three additional Cys residues in the mature protein and one in the signal peptide. Alanine substitution of Cys-227 and Cys-336, alone or together, has significantly reduced hydrolase activity in a substrate-dependent manner, while the other Cys mutants did not drastically affect activity (19).

The three-dimensional structures of the closest homologs of HLAL, HGL and dog gastric lipase (DGL), have been solved (20, 21). The structure of HGL (lid-closed form) consists of a core and a cap domain, which includes a “lid” region that covers the active site (20). Another feature that is common to all α/β hydrolase family members is the presence of a classical catalytic triad (Ser-153/His-353/Asp-324) and an oxyanion hole (20, 22). The displacement of the lid region is essential for the substrate to access the catalytic Ser-153 at the substrate binding site. Subsequently, the same group has published the so called “lid-open” conformation of DGL and proposed a binding mode for a TG, its natural substrate (21). Based on the crystal structure of the HGL, a homology model of HLAL was proposed (20).

In the present study, we have expressed and purified a triple glycosylation mutant (TGM) (N51Q, N80Q, and N300Q; mature protein numbering) of HLAL and determined the crystal structure at 2.6 Å resolution in its lid-closed conformation. Biochemical studies with wild-type and a lid deletion construct, where a small segment of the lid domain was removed, were performed with an objective to gain insights into the biological significance of the lid region. Molecular dynamic simulation studies with the full-length enzyme were performed to understand the cap domain movements under different physiological conditions. A structure-based molecular model speculating the loss of function of HLAL CESD mutant, H274Y, is also discussed.

## MATERIALS AND METHODS

### Cloning and expression of recombinant HLAL in insect cells

The DNA sequence encoding full-length wild-type HLAL (UniProt identification number P38571) was custom-synthesized from GeneArt gene synthesis (Thermo Fisher Scientific, Waltham, MA) with codons optimized for insect cell, *Spodoptera frugiperda*, expression. Codons for tobacco etch virus (TEV) protease cleavage site (ENLYFQG), linker (ASG), and FLAG-tag (DYKDDDDK) residues were added to the C terminus of the enzyme to facilitate purification and tag cleavage. The synthesized DNA [which also includes kozak sequence (GCCACC), ATG initiation codon, and stop codon] was subcloned into the baculovirus expression vector, pFastBac1 (Thermo Fisher Scientific), and the recombinant virus was generated using the BAC-TO-BAC expression system following the manufacturer’s instructions (Thermo Fisher Scientific). Oligonucleotide-mediated site-directed mutagenesis and deletion mutagenesis were performed using the QuikChange II site-directed mutagenesis kit according to the supplier (Agilent Technologies, Santa Clara, CA). The TGM was generated by substituting Asn-51, Asn-80, and Asn-300 with Gln (N51Q, N80Q, and N300Q). The lid deletion mutant (LDM) was constructed by deleting residues ^238^NLCFLLC^244^ from the mature protein.

Expression of wild-type and mutant enzymes was achieved by infecting insect cells (Sf-9) at a cell density of 2 × 10^6^ viable cells per milliliter with a multiplicity of infection of 0.5 in a serum-free insect cell medium, SF-900 III SFM (Thermo Fisher Scientific), as previously described (23). The culture supernatant that contained the secreted recombinant protein was filtered using a 0.2 μm filter and purified using anti-FLAG M2 affinity gel (Sigma-Aldrich Inc., St. Louis, MO) as previously described (23). The eluted fractions were pooled and concentrated to a final volume of 1.0 ml using Vivaspin® (10,000 MWCO) concentrator following the manufacturer’s instructions (Sartorius Stedim Biotech, Goettingen, Germany). The FLAG tag was removed by treating with TEV protease (New England Biolabs, Ipswich, MA) to a final concentration of 0.05 mg of TEV per 1.0 mg of HLAL protein at 4°C overnight. The recombinant HLAL was further purified by gel filtration chromatography using a Superdex-200 HiLoad 16/60 column (GE Healthcare, Boston, MA) in size exclusion chromatography (SEC) buffer [50 mM MES (pH 5.0), 150 mM NaCl, 5% glycerol, 1 mM TCEP, and 0.001% Triton X-100]. Protein concentrations were determined using either the BCA assay system (Pierce) with BSA as a standard or using absorbance at 280 nm using NanoDrop 2000C (Thermo Fisher Scientific). The samples were analyzed on SDS-10% PAGE.

### Enzymatic activity of recombinant HLAL

Lipase activity was monitored using a coupled enzyme system that uses the free cholesterol product of the reaction to generate a highly fluorescent compound, resorufin, via cholesterol oxidase and horseradish peroxidase as previously described (23). The plates were read using a SpectraMax microplate reader and the values were plotted using GraphPad Prism version 7 for Windows (GraphPad Software, La Jolla, CA).

### Fluorescence-based protein thermal stability assay

A temperature-based ThermoFluor assay with a SYPRO dye (Molecular Probes, Eugene, OR) was used to assess the stability of proteins at pH 5.0 as described previously (23). In brief, 50 μl of protein at 5 μM was mixed with 0.5 μl of 200-X SYPRO orange solution (Thermo Fisher Scientific) in a 96-well PCR plate and loaded into iCycler iQ real-time detection system (Bio-Rad, Hercules, CA) following the manufacturer’s instructions (Bio-Rad). The temperature was ramped from 25°C to 99°C in 1°C increments with a dwell time of 6 s. Excitation and emission wavelengths of SYPRO orange are 490 and 530 nm, respectively. The changes in intensity of fluorescence emission as a function of temperature were monitored. The negative first derivative of the fluorescence change (−dRFU/dT, where RFU is relative fluorescence unit) for each protein was plotted against temperature, and the melting temperature (*T_m_*) was defined as the minimum in the −dRFU/dT curve.

### Mapping of glycosylation sites by LC/MS

Site-specific glycosylation of the wild-type and mutant HLAL protein was analyzed using LC/MS techniques. Proteolytic digestion and PNGase treatment of the samples were performed as described previously (23). Peptides were separated by RP-HPLC on a 5 mm, 300 Å, 100 × 0.5 mm PROTO C18 column (Higgins Analytical, Inc., Mountain View, CA) using the following gradient: 1.6% B/2 min, 1.6–35% B/98 min, 35–80% B/10 min at 10 μl/min (A is 0.1% formic acid in water and B is 0.1% formic acid in acetonitrile). Mass spectra from *m/z* 400–2,000 were acquired in the LTQ Orbitrap XL mass spectrometer (Thermo Fisher Scientific) with the most abundant ions targeted for concurrent MS/MS in the linear ion trap with collision energy of 35%, 2 Da isolation widths, and dynamic exclusion of 3 for 30 s. Sequence database searches were performed using the Mascot algorithm with differential modifications including *S*-carboxyamidomethylation of Cys and deamidation of Asn for conversion of Asn to Asp from deglycosylation.

### Gel-based activity profiling with fluorophosphonate-rhodamine

Binding of the fluorophosphonate-rhodamine (FP-rho) probe (ActivX Biosciences, Inc., La Jolla, CA) to the protein was obtained by transferring 10 μg of purified enzyme to a microcentrifuge tube, which contained 0.100 ml of either PBS at pH 7.5 or 50 mM sodium acetate buffer at pH 4.7. FP-rho probe stock (100 μM) (Thermo Fisher Scientific) was added to each sample to a final concentration of 5.0 μM and mixed well. The samples were incubated at room temperature for 15–30 min. The reaction was stopped by adding 2× Laemmli reducing sample buffer and boiling for 5 min. The final samples were separated on SDS-10% PAGE and analyzed by fluorescent gel scanning (Image Lab, Bio-Rad) Western blotting method. The LAL band intensity was calculated using Lab version 5.2.1 software (Bio-Rad).

### Crystallization, data collection, and structure determination of HLAL

Crystals of HLAL-TGM were grown in 1.8 M (NH_4_)_2_SO_4_ and 0.1 M CH_3_COONa (pH 5.5) via sitting drop vapor diffusion at 12 mg/ml protein concentration. Rectangular crystals, to a size of 20 × 40 μm, were obtained from recombinant HLAL-TGM expressed in the insect cell/baculovirus expression system. Crystals were flash-cooled in liquid nitrogen after cryoprotection in a solution containing 3.2 M (NH_4_)_2_SO_4_, 5% glycerol, and 0.1 M CH_3_COONa (pH 5.5). Diffraction data were collected at the 17-ID beamline of the Advanced Photon Source of the Argonne National Laboratory on a Pilatus detector. X-ray data were processed using program XDS (24). The structure was determined by molecular replacement method, using Phaser (25) and the HGL structure [Protein Data Bank (PDB) code: 1HLG] as starting model. Structure was refined using the Buster (26) program and manual model building using Coot (27). Ramachandran statistics are 99.5% in preferred and allowed regions with 0.51% outliers. All outliers are at the loop containing Val-226 (between α9 and α10), where the electron density is not continuous, indicating the flexibility of the loop. The overall MolProbity (28) score is 2.41 and the clash score is 5.0. Figures were prepared with PyMOL (29). The coordinates and structure factor amplitudes have been deposited in the PDB with accession number 6V7N.

### Homology model of LIPA active form

The homology model of the active (lid-open) form of HLAL was built with Prime module within Maestro software package (Version 2014, Schrodinger Inc., New York, NY) using DGL (lid-open) X-ray structure (PDB code: 1K8Q) as a template. The final model was titrated at pH 4.5 and optimized with the retrained minimization of RMSD 0.30 Å.

### Molecular dynamics simulations

Molecular dynamics (MD) simulation studies for HLAL active (lid-open) and inactive (lid-closed) forms were carried out at pH 7.4 and pH 4.8 using Desmond program available in the Maestro software package. The input protein structures provided to Desmond were prepared using the Protein Preparation Wizard tool and titrated to the corresponding pH using ProPKA. The system was set up for simulation using a predefined TIP3P explicit solvent model in an orthorhombic box with periodic boundary as 10 Å buffer distance between the solute structures and the simulation box boundary. The system was neutralized by adding counter Cl^−^ ions. The simulation was performed under isothermal isobaric ensemble (NPT) for 500 ns with the pressure of 1.013 bar and the temperature of 26.85°C. The recording interval for each frame was 20 ps, resulting in a total of 25,000 recorded frames. All other parameters were set to default in Desmond. Protein root mean square fluctuation (RMSF) analysis was conducted using the Simulation Event Analysis tool within the Desmond program.

## RESULTS

### TMG did not adversely affect expression and biological activity of HLAL

**Fig. 1.**
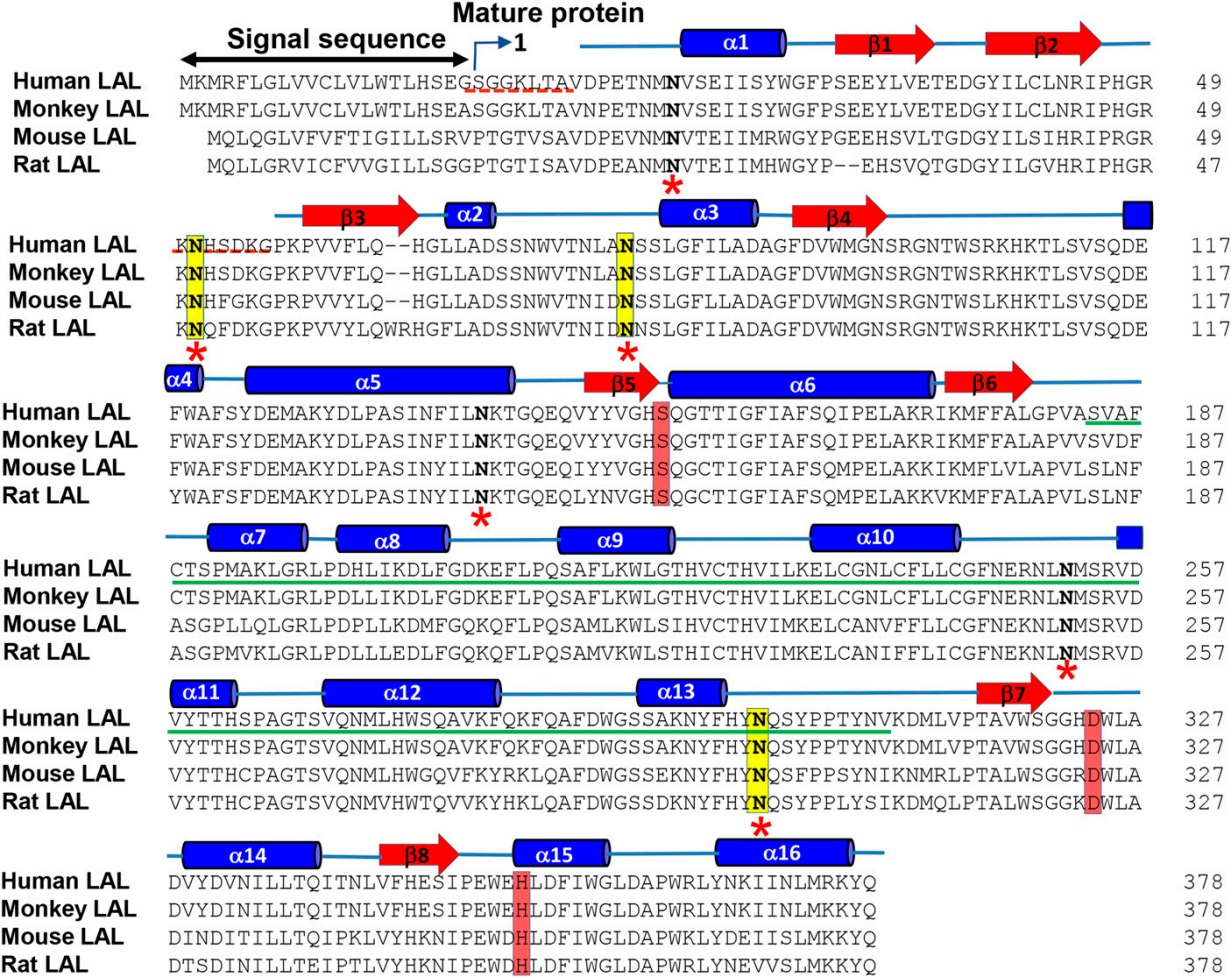
Sequence alignment of mammalian (human, monkey, mouse, and rat) LALs. Asn residues that are mutated to Gln (N51Q/N80Q/N300Q, mature protein numbering) in the TGM are displayed with yellow shading. The catalytic triad residues (Ser-153, Asp-324, His-353; mature protein numbering) are displayed with red shading. The secondary structures are indicated above the HLAL sequence. The cap domain sequences (residues 184–308) are underlined by a green line. Red dashed lines represent regions where there is no electron density. *Sites that are glycosylated in the wild-type HLAL.

Sequence alignment of LAL isoforms from human, monkey, mouse, and rat revealed five putative glycosylation sites (Asn-15, Asn-80, Asn-140, Asn-252, and Asn-300; mature protein numbering) that are highly conserved among all species, while a sixth site (Asn-51) is present only in human and monkey (**Fig. 1**). The overall amino acid identity among these isoforms with respect to HLAL are: monkey LAL, 98%; mouse LAL, 74%; and rat LAL, 76% (Fig. 1). We have eliminated three out of the six glycosylation sites by a site-directed mutagenesis approach and generated a TGM. In the TGM, residues Asn-51, Asn-80, and Asn-300 (mature protein numbering) were converted into Gln (N51Q/N80Q/N300Q). The wild-type and the TGM proteins were expressed in insect cells (Sf9) and purified from the culture media using anti-FLAG M2 antibody affinity resin by batch binding method. The expression levels and final yields of the wild-type and TGM were comparable (1–2 mg/l). In SEC, the elution profile of wild-type and TGM looked similar (see supplemental Fig. S1). In both cases, the retention time of the major peak appeared to be corresponding to monomeric LAL (∼44 kDa). However, in SDS-PAGE gel (supplemental Fig. S1, inset), the wild-type protein migrates slightly higher than the TGM.

MS analysis of insect cells that expressed wild-type recombinant HLAL showed that all six potential N-glycosylation sites were occupied by high mannose N-linked oligosaccharides (**Table 1**). The percent glycosylation on each site was quantitated by subjecting the samples to proteolytic digestion and PNGase treatment (see supplemental Fig. S2) as described in the Materials and Methods. As shown in Table 1, there is no significant difference in the pattern of glycosylation between the wild-type and the TGM at the nonmutated sites, Asn-15, Asn-140, and Asn-252 (Table 1). No modifications were observed at the mutated sites, N51Q, N80Q, and N300Q.

**TABLE 1.** Comparison of glycosylation pattern between wild-type and glycosylation mutants

Glycosylation Site	Wild-Type		Glyco-Mutant	
	Glycosylation (%)*^a^*	Major Glycans	Glycosylation (%)*^a^*	Major Glycans
Asn-15	100	Man3F	100	Man3F
Asn-51	78	Man3, Man3F	Mutated	Mutated
Asn-80	100	Man3F	Mutated	Mutated
Asn-140	66	Man3, Man3F	66	Man3, Man3F
Asn-252	>95	Man7, Man8, Man9	>95	Man6, Man7
Asn-300	100	Man3F	Mutated	Mutated

Man (oligomannose) F (fucose). In general, proteins expressed in insect cells contain high mannose N-linked oligosaccharides with varying amount of mannosyl residues; Oligoman-3 contains 3 mannosyl residues. Amino acid number corresponds to the mature protein. Man, oligomannose; F, fucose.

a Approximate estimate.

**Fig. 2.**
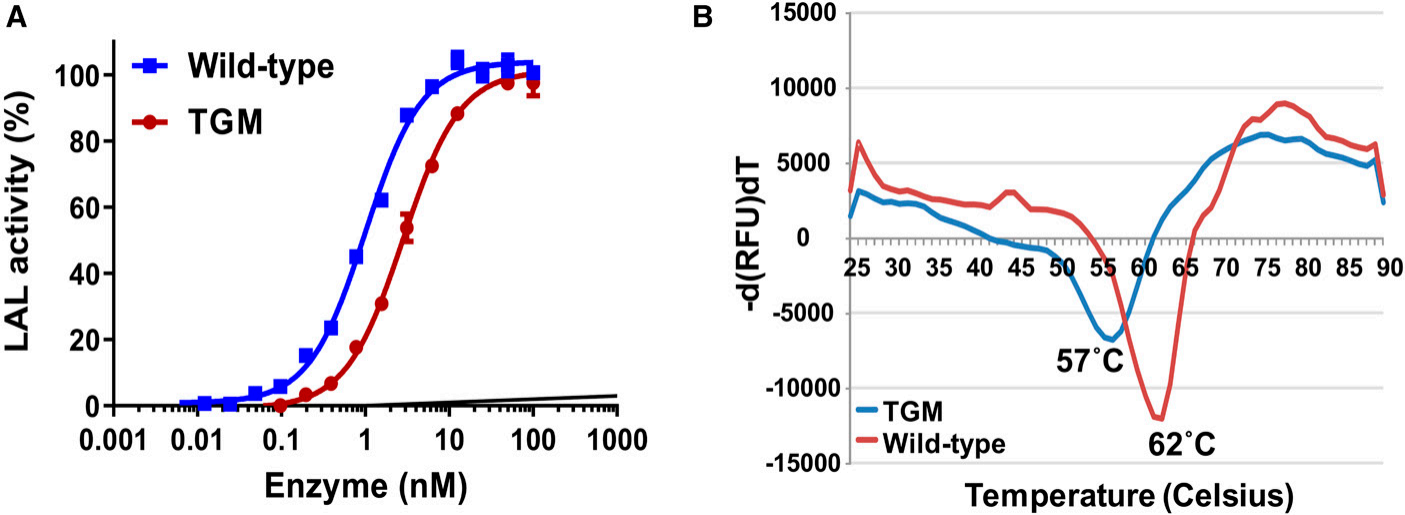
Functional properties of recombinant wild-type and TGM. A: LAL-dependent hydrolysis of cholesterol oleate to free cholesterol was measured using an Amplex Red-coupled assay as described in the Materials and Methods. B: Thermal unfolding profile of wild-type (red) and TGM (blue) proteins using SYPRO orange as the reporter dye. The estimated *T_m_* values are shown in the graph.

Enzymatic activity of the SEC pool was evaluated using a coupled enzymatic assay (see the Materials and Methods). As shown in **Fig. 2A**, both wild-type and TGM showed comparable enzymatic activity and the estimated EC_50_ values (effective concentration of enzyme required to achieve 50% of the maximum attainable hydrolysis of cholesteryl oleate as substrate under these experimental conditions) were 1.002 ± 0.1 and 2.819 ± 0.2 nM, respectively. Next, we evaluated the thermal stability of the wild-type and TGM proteins using a fluorescence-based (SYPRO) thermal unfolding assay, which distinguishes folded and unfolded proteins through recognition of exposed hydrophobic areas as a function of increasing temperature. As shown in Fig. 2B, at pH 5.0, the wild-type protein undergoes a major unfolding event at 62°C (*T_m_*), whereas the *T_m_* value for the TGM is slightly shifted left, 57°C.

**Fig. 3.**
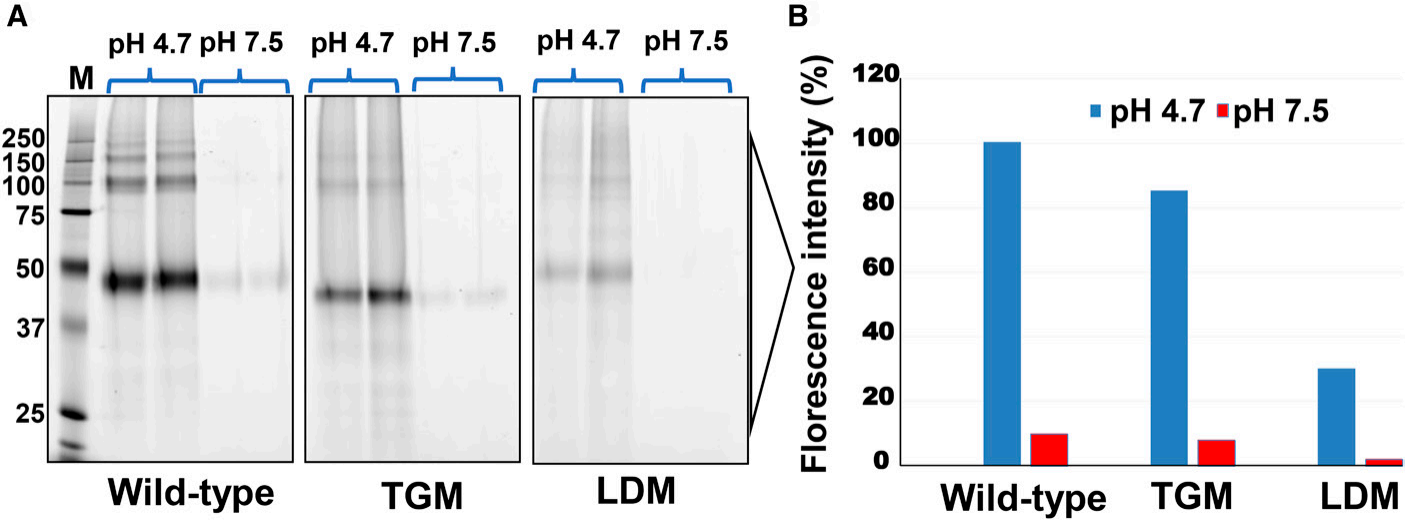
Gel-based activity profiling of recombinant proteins with FP-rho. SDS-10% PAGE indicating the probe binding at pH 4.7 and 7.5. The arrow shows the LAL band. Each bar in the right panel represents the mean of the relative intensity of the LAL band. The wild-type LAL band intensity is considered as 100%.

Furthermore, we evaluated the specific binding of an activity-based chemical probe (ABP), FP-rho, on purified wild-type and TGM proteins by direct monitoring of its interaction with the active site at pH 4.7 and 7.5 in a gel-based assay. As shown in **Fig. 3**, both wild-type and TGM proteins reacted with the probe closer to its physiological pH (pH 4.7). Consistent with the activity and the *T_m_* data, the HLAL band intensity of the mutant (TGM) is about 14% lower than that of the wild-type (Fig. 3). However, the interaction of the probe with the purified protein was significantly affected at pH 7.5 in both wild-type and TGM (Fig. 3).

### HLAL adopts a well-defined canonical structure of α/β hydrolase superfamily

**Fig. 4.**
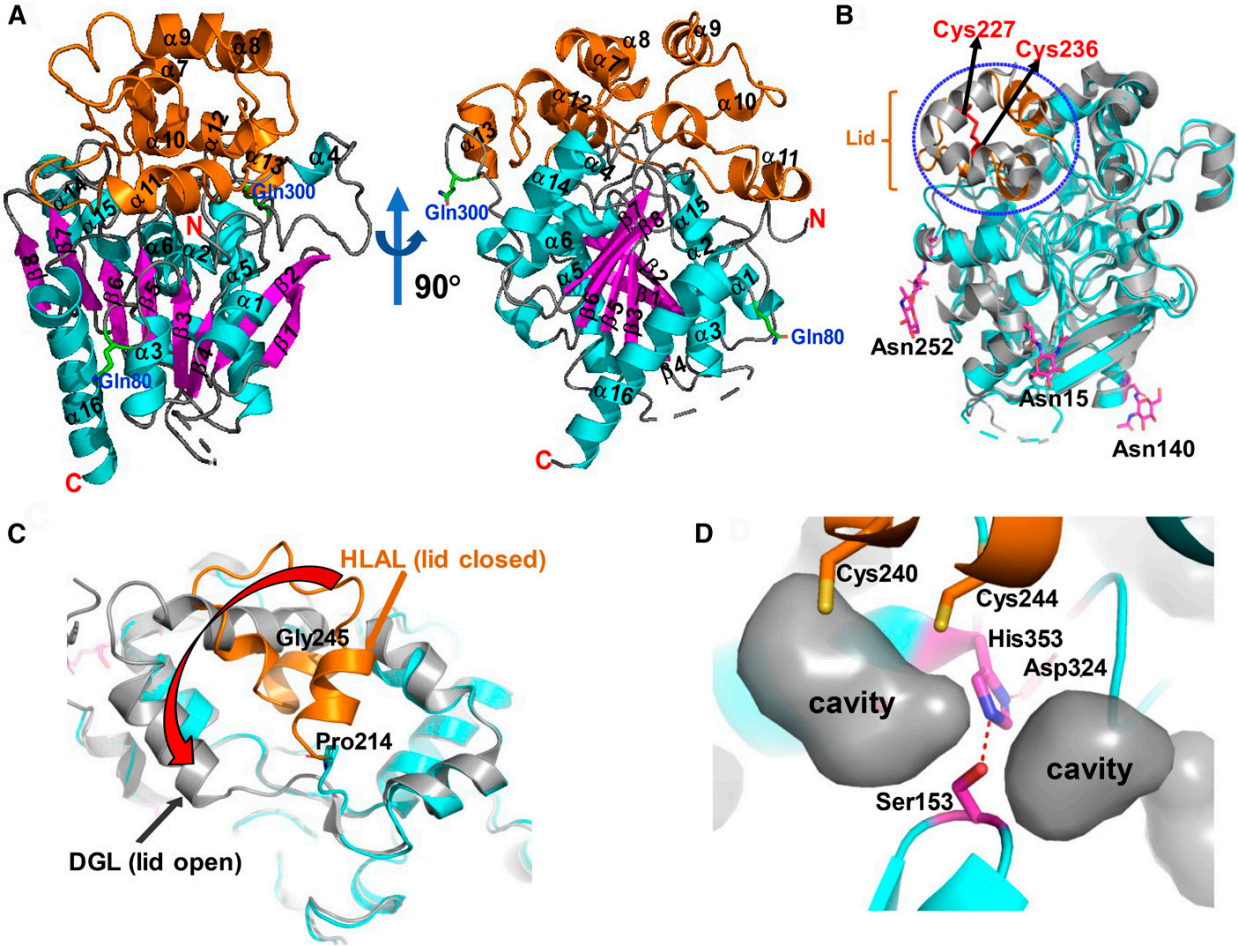
The structure of HLAL lid-closed form. A: Overall structure displaying the core and cap domains. Polypeptide is depicted as a ribbon with helices of the core domain colored cyan and β-strands magenta, helices of the cap domain are colored orange. Mutated glycosylation residues (N80Q, N300Q) are indicated (N51Q is disordered). Dotted line indicates the disordered region. B: Overlay between HLAL (cyan) and HGL (gray) structures. The lid residues are circled by blue dotted lines and the lid residues of HLAL are shown in orange. Disulfide bonds between Cys-227 and Cys-236 are indicated in red. Asn-attached sugars are shown in ball and stick form. C: An overlay between the lid domains of DGL [lid-open form (gray)] and HLAL [lid-closed form (cyan and orange)]. The red arrow indicates the movement of the lid from closed to open form. The hinge residues, Gly-245 and Pro-214, are shown. D: Close-up of the active site illustrating the catalytic triad residues. Cys-240 and Cys-244 are visible above the catalytic triad. Cavities between the active-site and the lid residues are shown.

The recombinant HLAL-TGM was crystallized in the orthorhombic space group P2_1_2_1_2 with two molecules in the asymmetric unit and diffracted to around 2.6 Å. The final “lid-closed” structure of HLAL was refined to 2.6 Å resolution using restrained noncrystallographic symmetry between the two molecules. Ramachandran statistics are 99.5% in preferred and allowed regions with 0.51% outliers. The crystallographic statistics for the final model are shown in supplemental Table S1. The crystal structure includes residues 9–49 and 57–384 and five additional residues, ASENL, at the C-terminus end, which were part of the TEV protease cleavage site added for cleaving the FLAG tag. As with HGL, HLAL consists of a globular domain that belongs to the α/β hydrolase fold family. The overall architecture consists of a core domain and a cap domain (**Fig. 4A**). The core domain, which constitutes residues 9–183 and 310–384 (mature protein numbering), contains a central β-sheet composed of eight strands (seven parallel) and six helices, three on each side of the β-sheet (Fig. 4A). The cap domain fold (residues 184–309) is a complex mixture of eight helices, turns, and random coils, including a lid (residues 215–244) that adapts a “closed” conformation (Fig. 4A). Least-squares Cα superposition of the lid-closed conformation of HGL and HLAL reveals the similarity and yields an overall RMSD value of 0.55 Å for 294 Cα atom pairs. The difference in the Cα backbone is more pronounced in the cap region (residues 215–244) with an RMSD of 2.7 Å (Fig. 4B). As shown in Fig. 4B, the structure also includes GlcNAC sugar residues on three of the N-glycosylation sites (Asn-15, Asn-140, and Asn-252); the other three potential sites (Asn-51, Asn-80, and Asn-300) were mutated to Gln. No electron density for the fucose residues that were attached at GlcNAc, as per the MS analysis, was visible in the structure. The amino acid sequence of the matured and processed LAL contains six Cys residues (Cys-41, Cys-188, Cys-227, Cys-236, Cys-240, and Cys-244). Cys-227 and Cys-236 are involved in a disulfide bridge and the free Cys were unambiguously assigned (Fig. 4B).

An overlay of the lid region between the lid-open form (DGL) and HLAL (lid-closed form) is shown in Fig. 4C. Two key residues, Pro-214 and Gly-245, act as the hinge to allow the lid to move between the open and closed forms. Several cavities are observed at the interface between the active site and the core domain (Fig. 4D).

### Role of Asp-361 in HLAL activation at low pH

**Fig. 5.**
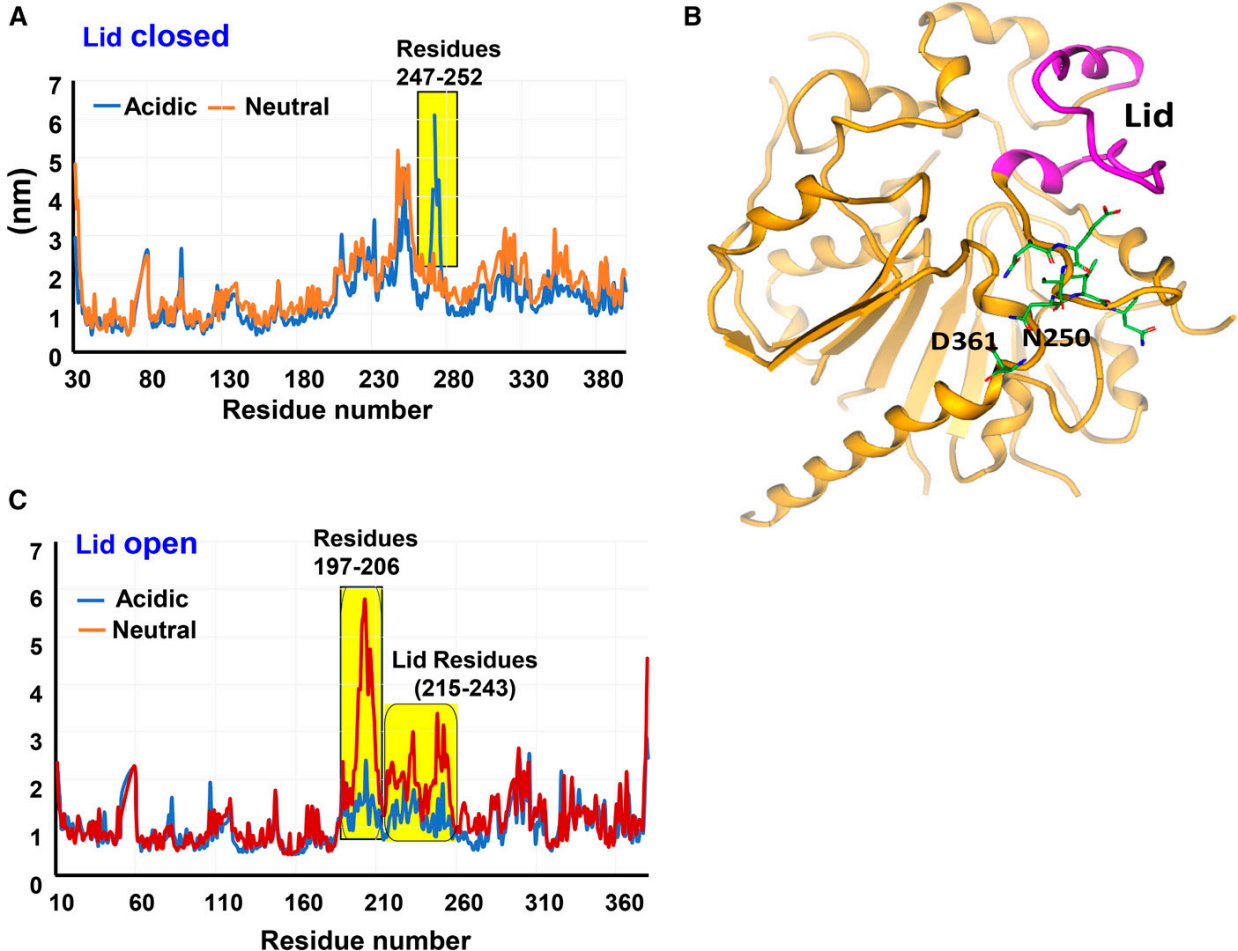
RMSF plot of HLAL at acidic and neutral pHs. A: Overlay of closed form at neutral and acidic pHs. B: HLAL structure (closed) illustrating atomic interaction of Asn-250 with Asp-361. The lid region is shown in pink. C: Overlay of open form at neutral and acidic pHs. Yellow highlights represent the differences in mobility.

MD simulation studies were performed on the lid-closed (inactive) and lid-open (active) forms of HLAL at pH 7.4 and 4.8. The lid-closed simulated structure showed significant fluctuation between residues Arg-247 and Asn-252 at acidic pH in comparison with the neutral pH (**Fig. 5A**). The residues that showed substantial fluctuation are illustrated at the structure of HLAL (Fig. 5B). The RMSF plot of the lid-open form showed a different profile. At the neutral pH (pH 7.4), the residues between 197 and 243 (cap region) showed much higher fluctuation than that at acidic pH (Fig. 5C).

### Deletion of residues 238–244 from the lid domain leads to soluble aggregation, loss of enzymatic activity, and reduced binding to ABP

**Fig. 6.**
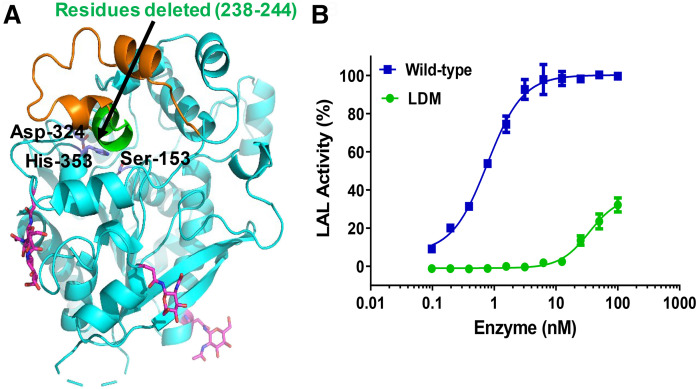
LDM construct. A: HLAL structure showing the helix 11 residues (^238^NLCFLLC^244^) that are deleted from the lid region (green). The lid residues are colored orange. The catalytic residues at the active site are indicated. B: Comparison of the LAL-dependent hydrolase activity of wild-type and the LDM.

We have generated a LDM by deleting lid residues ^238^NLCFLLC^244^, helix α11, of the mature protein (**Fig. 6A**). The expression levels and final yields of LDM were comparable to those of the wild-type (1–2 mg/l). As shown in Fig. 6B, the catalytic activity of LDM was significantly affected as compared with the wild-type protein. The estimated EC_50_ values for the wild-type and LDM were 0.92 ± 0.1 nM and 34.38 ± 8.0 nM, respectively. Separation of LDM protein on a SEC column displayed a significantly different elution profile than that of the wild-type (see supplemental Fig. S1). On the SEC column, the major peak of LDM eluted just after the void volume corresponds to high molecular mass aggregates (∼650 kDa). In comparison, LDM did not show any significant peak corresponding to the elution profile of monomeric wild-type protein (supplemental Fig. S1). We also examined the binding of the ABP to purified LDM protein at pH 4.7 and 7.5 in a gel-based assay. As shown in Fig. 3, the wild-type protein showed significant binding at pH 4.7, whereas LDM showed only weak reactivity [25% LAL band intensity compared with the wild-type (100%)]. Both the wild-type and LDM proteins were not very reactive with the probe at pH 7.5 (Fig. 3).

### Substitution of His-274 with Tyr escalates lid movement

**Fig. 7.**
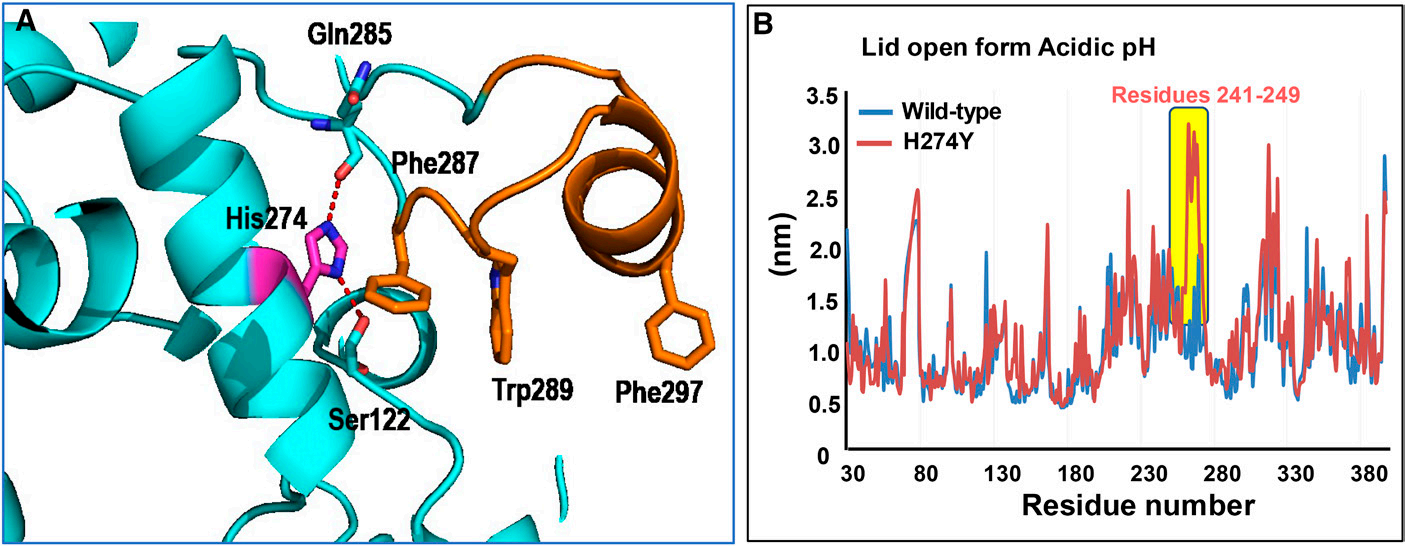
HLAL structure displaying the H274Y interactions. A: HLAL interactions with the cap domain residues. B: Comparison of wild-type and H295Y mutant (lid open) RMSF plot at acidic pH. Yellow highlight represents the differences in mobility.

Structural analysis of HLAL showed that His-274 is involved in hydrophobic interactions with Ser-122 and Gln-285 of the core domain (**Fig. 7A**). We have performed MD simulation on the structure of HLAL wild-type and H274Y mutant at an acidic pH (pH 4.5) and evaluated the dynamics of structural movements. The RMSF graph showed that H274Y exhibited high flexibility at the putative lid residues 241–249 in comparison with the wild-type HLAL under similar conditions (Fig. 7B).

## DISCUSSION

LAL is an important enzyme for the regulation of cholesterol homeostasis. Unlike the well-known mammalian lipases of the lipase superfamily, triacylglycerol lipases, LAL shows a dual catalytic activity, capable of hydrolyzing both TG and CE. LAL deficiency in humans results in multisystem autosomal recessive disease phenotypes, WD and CESD. Altered LAL function has also been implicated in the development of atherosclerosis in the population at large (30, 31). Structural data for HGL (lid-closed form) and DGL (lid-open form) in complex with undecyl butyl (C11Y4) phosphonate inhibitor have been elucidated (20, 21). However, the molecular mechanisms leading to loss of function of these missense and nonsense homozygous single point mutations in the general population remain poorly understood. In this study, we solved the first apo crystal structure of HLAL at 2.6 Å resolution in its lid-closed form, compared it with previous crystal structures of HGL and DGL, and discussed the possible role of the highly variable cap/lid domain in regulating the catalytic site.

Numerous crystal trials with the wild-type and enzymatically deglycosylated HLAL protein were unsuccessful. We speculated that the posttranslational heterogeneity could be restraining the crystal growth. Oligosaccharide residues are of particular relevance for these enzymes because phosphorylation of mannose residues mediates the targeting of these hydrolases to the lysosome via mannose-6-phosphate receptors (32). Human LAL has six putative N-glycosylation sites belonging to the classical N-glycosylation motif N-X-S/T. MS analysis of wild-type HLAL expressed in insect cells showed the presence of oligomanose-3 and fucose as the major glycans attached to GlcNAc at all six putative glycosylation sites (Table 1). Zschenker et al. (17) investigated the function of oligosaccharide side chains of LAL using systematic mutagenesis of all potential glycosylation sites. They revealed that glycosylation at Asn-140 and Asn-252 are critical for LAL activity, whereas mutations of Asn-51, Asn-80, and Asn-300 have no significant effect on LAL activity compared with the wild-type. Based on this data, we have generated a TGM by converting Asn-51, Asn-80, and Asn-300 into Gln (N51Q/N80Q/N300Q). A previous study has shown that suppression of all N-linked glycosylation of LAL using tunicamycin, a glycosylation inhibitor, had a major impact on enzymatic activity and secretion of the enzyme to the media of the cultured cells (18). However, our results showed that the elimination of just three N-linked glycosylation sites did not significantly affect protein expression levels, secretion to the media, enzymatic activity, SEC profile, and glycosylation patterns. Nonetheless, TGM exhibited marginal influence on the biological activity (Figs. 2A, 3) and thermal stability of the protein at pH 5.0 (Fig. 2B). Recombinant HLAL-TGM protein yielded cubic crystals and enabled us to solve the first apo structure of HLAL. HLAL has high structural similarities with the crystal structure of HGL. Nevertheless, there are significant structural changes between HGL and HLAL at the cap domain where there are substantial amino acid sequence disparities (see supplemental Fig. S3). Consistent with the HGL structure (20), the electron density starts from residue 9 and lacks visible density for residues from 50 to 56 as well. However, these disordered loops were visible in the structure of DGL lid-open structure. It has been suggested that the N-terminal segment is stabilized by contacts with the open lid conformation, while the loop residues 54–56 were stabilized by packing interactions (21). As is typical of the α/β hydrolase fold, the nucleophilic Ser, which is part of the Ser-153/His-353/Asp-324 catalytic triad, is located at an extremely sharp turn called the “nucleophilic elbow,” between a central β-strand (β5) and a helix (α11). Asp-324 is located on a second sharp turn between β6 and α-10 and is involved in hydrogen bonding interactions with Asp-324 and His-353, which helps to position the imidazole to interact with the hydroxyl of Ser-153. Like the lid-closed conformation of HGL, several cavities were observed predominantly at the interface between the core and cap domains in HLAL structure. At the active site, a small cavity on top of the catalytic Ser-153 is formed by residues Asn-73, His-152, Leu-354, Leu-357, Trp-358, and two Cys residues (Cys-240, Cys-244) and contains a water molecule, which forms hydrogen bonds with the carboxyl backbone of Leu-67. Another cavity near the active site is mainly formed by hydrophobic residues, Leu-67, Val-182, Met-192, Trp-275, and Leu-326. These two cavities could potentially provide solvent with access to the catalytic Ser-153 (Fig. 4D).

The cap domain fold (residues 184–308) is a complex mixture of eight helices (α7 to α14), turns, and random coils. It contains a lid (residues 215–244) that adapts a closed conformation in this structure. Many water molecules, predominantly at the interface between cap and core domains, were observed (data not shown). The lid region is formed by an α-helix and two short strands that covers the active site. As suggested before, Pro-241 and Gly-245 residues flank the lid acting as hinges, with the *trans*-*cis*-proline isomerization inducing opening and closing of the lid (21). It is apparent from the structure that the catalytic Ser-153 is completely inaccessible and substantial conformational changes must take place before substrate can bind in the postulated active site. The repositioning of this loop could be necessary to render access to the substrate. The closed lid of HLAL is made of three short helices (α9, α10, and α11), whereas the open form of the lid is composed of two helices (21). Despite the overall structural similarity between the lid-closed HGL and HLAL, super positioning of the Cα backbones of the lid region displayed significant structural differences, an RMSD of 2.7 Å (Fig. 4B). Furthermore, in human pancreatic lipase, colipase (a small protein cofactor), interacts with the C-terminal noncatalytic domain of lipase, thereby stabilizing an active conformation and considerably increasing the catalytic activity (33, 34). However, such a cofactor for HLAL has not been discovered to date.

The structure also included electron density for GlcNAc sugar residues on three of the N-glycosylation sites (Asn-36, Asn-161, and Asn-273), the other three potential sites (Asn-72, Asn-101 and Asn-321) were mutated to Gln (Fig. 5A). The protein sequence of HLAL contains six Cys residues in the mature protein compared with three Cys residues in HGL. Among these, Cys-227 and Cys-236 (mature protein numbering) form a disulfide bridge (Cys-227–Cys-236), whereas the other Cys residues are free, as previously discussed in the case of HGL. Cys-240 and Cys-244 are in the lid region and are deeply buried pointing toward the catalytic Ser-153 (Cys-244 is only 5 Å away from catalytic Ser-153). Removal of the putative lid region (residues 215–244) from the structure exposes a large hydrophobic surface around the active site, suggesting a conducive environment for lipid substrate binding. This was supported by the lid-open structure of the DGL (21). Besides single point mutations (T267I, Q64R, L273S, G66V, and H274Y), large deletions of residues 205–253 (exon 7) and residues 254–277 (exon 8) in the cap domain have been associated with CESD (35, 36). These deletion mutants retained only residual enzymatic activity (35). In this study, we have generated a LDM by deleting a small seven amino acid helix (α11), ^238^NLCFLLC^244^, that is covering the active site, and evaluated its functional role in regulating the catalytic site. We speculated that the elimination of the lid would provide better access to the substrate and may lead to higher catalytic activity. But surprisingly, the catalytic activity of LDM was significantly reduced compared with the wild-type (>3,000-fold reduction, Fig. 6B) in our coupled enzyme system assay. The SEC profile indicated that the majority of LDM proteins formed soluble aggregates and eluted around the void volume (>610 kDa); whereas, the wild-type protein eluted later corresponding to its monomeric molecular mass (see supplemental Fig. S1). This suggests that the loss of activity of LDM is attributable to the enhanced self-aggregation or defective folding of the protein, possibly through its exposed highly hydrophobic active site.

Additionally, we used an ABP to validate the active site conformation of LDM in comparison with wild-type and TGM. ABPs achieved great importance in studying enzyme activities/active site conformation with classes of enzymes such as the serine hydrolases and metalloproteases (36). ABPs distinguish between active and inactive enzymes, often by a mechanism-based reaction with a reactive electrophile, which attacks the catalytic site to arrest the active state of an enzyme. We have used FP-rho, which covalently links to the nucleophilic residue in the active site of the enzyme only in an active conformation. Consistent with our activity data, wild-type and TGM proteins were highly reactive to the probe at pH 4.7; whereas, LDM protein showed only marginal reactivity at pH 4.7 (Fig. 3). As expected, none of these proteins were reactive at neutral pH, pH 7.5 (Fig. 3). This suggests that the hydrophobic active site is regulated by the lid region and its removal leads to an inactive self-aggregated form in the absence of lipid substrate. It is quite possible that the lid opening and substrate binding might happen simultaneously in order to protect the enzyme from self-aggregation and inactivation.

MD simulations have been used to gain insights into domain movements and function of biomolecules (37). To understand the HLAL pH activation mechanism, we performed MD simulation of HLAL lid-closed and -open forms (homology model) at both acidic (pH 4.8) and neutral (pH 7.4) pH. At acidic pH in the closed form, a key hydrogen bond interaction between Asn-247 and Asp-361 is disrupted. This leads to a high fluctuation (destabilization) of residues Asn-247 to Asn-252. Asn-250 is located at a loop (between α11 and α12) that abuts the lid and is in close proximity to the core domain residue Asp-361 (Fig. 5B). We speculate that, at neutral pH, Asp-361 is deprotonated and forms a hydrogen bond with Asn-250 and stabilizes the lid in a closed form. Whereas, at acidic pH, Asp-361 is protonated, no longer engaged in hydrogen bonding with Asn-250, and leads to the movement of the lid into an open (active) conformation. Due to the limitations of the MD simulation time, we do not observe a full lid-open conformation of the inactive form at the acidic condition. As a comparison, at neutral pH in the open form, a significant fluctuation is observed between Arg-197 and Leu-206 that leads to high fluctuation of the extended lid region (Fig. 5C). Interestingly, our HLAL MD simulation data is considerably different from that of an earlier study on HGL and DGL (38). MD simulations of HGL (at an acidic and neutral condition) and DGL (at acidic pH) showed maximum mobility in the lid residues 215–234, irrespective of the pH conditions (acidic/neutral) and lid-open (DGL) or lid-closed (HGL) forms (38). Besides the overlapping flexibility regions (residues 215–234), certain other regions from the core domain also showed fluctuations in HGL (38). However, in HLAL, we did not observe any additional fluctuations other than the lid/cap residues.

We have previously reported that mutating His-274 to Tyr (H274Y, mature protein numbering), the most common loss-of-function mutant identified in CESD phenotype, resulted in significant loss of lipase activity in addition to aggregation and defective protein folding (23). We speculated that H274Y would disrupt the hydrogen bond interaction between cap and core domains and thereby destabilize the protein. The complementary surface part of the lid domain facing the hydrophobic substrate binding site in the closed form is critical for maintaining the enzyme in an inactive conformation, and any subtle changes in that interaction can destabilize the enzyme. In our HLAL structure, His-274 in the α13 helix is involved in a hydrogen bond interaction with Ser-122 and Gln-285, is sandwiched by two aromatic residues (Tyr-123 and Phe-287), and stabilizes the interaction between the α13 helix and a long loop-helix-loop region (residues 286–304). Furthermore, we ran a MD simulation of the H274Y mutant at pH 4.5 and overlaid the RMSF plot to that of the wild-type (Fig. 7). The results suggested that substitution of His-274 with tyrosine significantly enhanced the flexibility to part of the lid region (Phe-241 to Arg-249). We speculate that H274Y interrupts potential interactions between the lid and core domains and eventually moves the lid away from the active site, thus leading to self-aggregation by exposing hydrophobic residues such as Phe-287, Trp-289, and Phe-297. However, further site-directed mutagenesis studies may help to substantiate our speculations. This structural information of HLAL along with MD simulation speculations may help to develop pharmacological agents that either rescue trafficking or folding defects or increase the functional activity of LAL, providing a path to an effective therapeutic option for WD and CESD patients.

### Data availability

All data are contained within the article. The coordinates and structure factor amplitudes have been deposited in the Protein Data Bank (PDB) with accession number 6V7N.

## Supplementary Material

Supplemental Data
