# Supplementary material for: Crystal structure of human lysosomal acid lipase and its implications in cholesteryl ester storage disease
Source: J Lipid Res. 2020 Jun 1;61(8):1192–202. doi: 10.1194/jlr.RA120000748 (PMC7397744; doi:10.1194/jlr.RA120000748)
Supplement: Supplemental Data [file supp_61_8_1192__index.html]

Crystal Structure of human Lysosomal Acid Lipase and its Implications in Cholesteryl Ester Storage Disease (CESD) — Crystal structure of human lysosomal acid lipase — Crystal structure of human lysosomal acid lipase and its implications in cholesteryl ester storage disease — Supplemental Data 

# Crystal structure of human lysosomal acid lipase and its implications in cholesteryl ester storage disease

## Supplemental Data

- Crystal Structure of human Lysosomal Acid Lipase, and its Implications in Cholesteryl Ester Storage Disease (CESD) - Supplemental data
