# Supplementary material for: Crystal structure of human lysosomal acid lipase and its implications in cholesteryl ester storage disease
Source: J Lipid Res. 2020 Jun 1;61(8):1192–202. doi: 10.1194/jlr.RA120000748 (PMC7397744; doi:10.1194/jlr.RA120000748)
Supplement: Supplemental Data [file supp_RA120000748_159367_2_supp_532523_qvjlv7.pdf]

Supplemental Table S1: X-ray crystallographic statistics for structural determination. Overall statistics of native data set for LIPA structural determination. Parenthesis represent the highest resolution shell.

**Data collection**	**LIPA**
Resolution (Å)	84.78 - 2.62 (2.667 – 2.622)
Wavelength	1.0
# of Observations	193147 (9632)
# of Unique Reflections	30000 (1463)
$CC_{1/2}$	0.996 (0.684)
R_{pim}	0.058 (0.471)
CC*	0.999 (0.924)
Space Group	$P2_12_12$
Unit Cell	
a,b,c (Å)	97.1, 165.5, 60.6
α,β,γ (°)	90.0 90.0, 90.0
Completeness (%)	100.0 (99.9)
Redundancy	6.4 (6.6)
R_{meas} (%)[a]	15.8 (122.0)
Mean ((I)/σ(I))	13.4 (2.8)
**Refinement**	
Resolution (Å)	84.78 – 2.62
Reflections (total/free)	29951 / 1518
Cutoff for Refinement	F > 0σ
R_{work}/R_{free} [a], %	18.5 /22.2
Atoms total/protein	6242/5973
Root mean square deviations	
Bonds (Å)	0.009
Angles (°)	1.1
Average B Factors (Å^2)	
Amino Acids	59.6
Heterogen	88.8
Waters	52.8
Wilson B Factors (Å^2)	65.4
Ramachandran plot	
Favored (%)	99.2
Allowed (%)	0.3
Outliers (%)	0.5
Clashscore	5.0

Footnote: R_{meas}, overall measure of error between multiple measurements of a reflection within I+/I-, independent of redundancy.

a R_{free} is the R_{work} based on 5% of the data excluded from the refinement.

$$R_{meas}=\frac{\sum_{hkl}\sqrt{\frac{n}{n-1}}\sum_{j=1}^{n}|I_{hklj}-<I_{hkl}>|}{\sum_{hkl}\sum_{j}I_{hklj}}$$

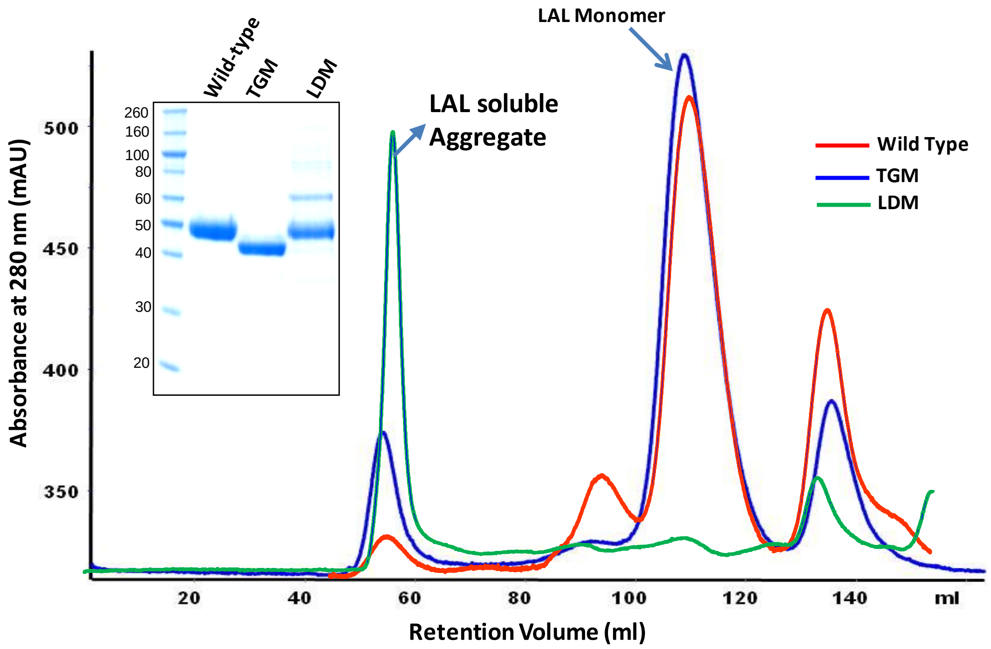

Supplemental Figure S1. Representative chromatogram of size-exclusion chromatography of recombinant HLAL on a superdex-200 HiLoad 16/60 column. Elution profiles of wild-type (red), triple glycosylation mutant (TGM, blue) and lid deletion mutant (LDM, green) are indicated. The retention time of LAL monomer and soluble aggregate are indicated. ***Inset***: Coomassie blue-stained SDS-10% PAGE analysis of the major peak sample collected from the column. Each lane contains 3.0 μg of protein (absorbance at 280 nm using NanoDrop 2000C).

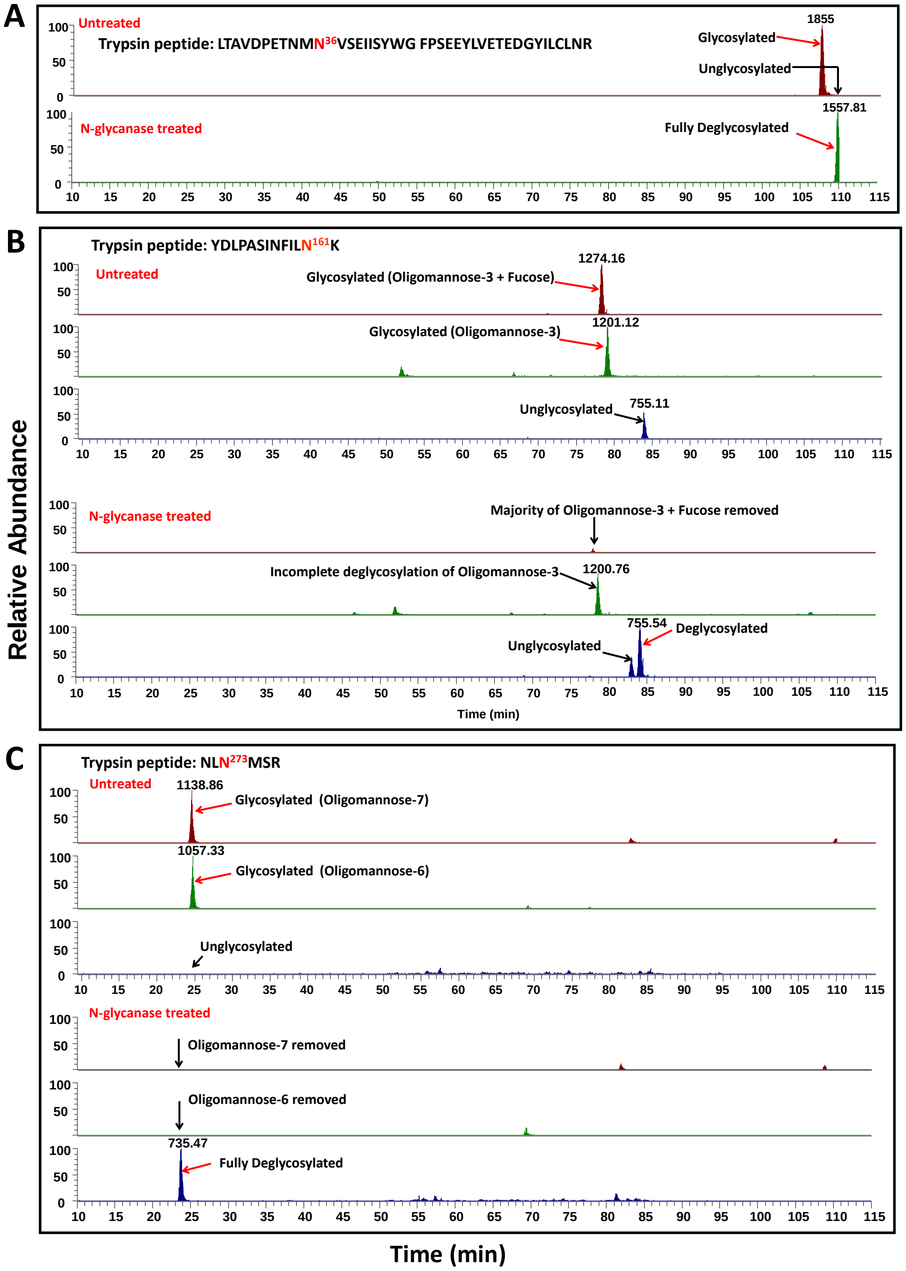

Supplemental Figure S2. Mapping of glycosylation sites by Liquid Chromatography/Mass Spectrometry

```
HLAL            3 GKLTAVDPETNMNVSEIISYWGFPSEEYLVETEDGYILCLNRIPHGRKNHSDKGPKPVVF
HGL             3 GKLHPGSPEVTMNISQMITYWGYPNEEYEVVTEDGYILEVNRIPYGKKNSGNTGQRPVVF
                  ***    **  ** *  * *** * *** * *******  **** * **    *  ****

HLAL           63 LQHGLLADSSNWVTNLANSSLGFILADAGFDVWMGNSRGNTWSRKHKTLSVSQDEFWAFS
HGL            63 LQHGLLASATNWISNLPNNSLAFILADAGYDVWLGNSRGNTWARRNLYYSPDSVEFWAFS
                  *******   **  ** * ** ******* *** ******** *     *    ******

HLAL          123 YDEMAKYDLPASINFILNKTGQEQVYYVGHSQGTTIGFIAFSQIPELAKRIKMFFALGPV
HGL           123 FDEMAKYDLPATIDFIVKKTGQKQLHYVGHSQGTTIGFIAFSTNPSLAKRIKTFYALAPV
                   ********** * **  **** *  ****************  * ****** * ** **
                                                                Lid
HLAL          183 ASVAFCTSPMAKLGRLPDHLIKDLFGDKEFLPQSAFLKWLGTHVCTHVILKELCGNLCFL
HGL           183 ATVKYTKSLINKLRFVPQSLFKFIFGDKIFYPHNFFDQFLATEVCSREMLNLLCSNALFI
                  * *    *   **   *  * *  **** * *   *   * * **    *  ** *  *

HLAL          243 LCGFNERNLNMSRVDVYTTHSPAGTSVQNMLHWSQAVKFQKFQAFDWGSSAKNYFHYNQS
HGL           243 ICGFDSKNFNTSRLDVYLSHNPAGTSVQNMFHWTQAVKSGKFQAYDWGSPVQNRMHYDQS
                   ***   * * ** ***  * ********* ** ****  **** ****   *  ** **

HLAL          303 YPPTYNVKDMLVPTAVWSGGHDWLADVYDVNILLTQITNLVFHESIPEWEHLDFIWGLDA
HGL           303 QPPYYNVTAMNVPIAVWNGGKDLLADPQDVGLLLPKLPNLIYHKEIPFYNHLDFIWAMDA
                   ** ***  * ** *** ** * ***  **  **    **  *  **   ******  **

HLAL          363 PWRLYNKIINLM
HGL           363 PQEVYNDIVSMI
                  *   ** *
```

Supplemental Figure S3. Sequence alignment of human lysosomal acid lipases (HLAL) and human gastric lipase (HGL). The “cap” region is displayed with yellow shading. The “lid” region is marked with a red line above the sequence.

* Residues that are identical
